# Low-input CUT&Tag for efficient epigenomic profiling of zebrafish stage I oocytes

**DOI:** 10.3389/fcell.2024.1475912

**Published:** 2024-12-04

**Authors:** Qianwen Zheng, Xiaotong Wu, Xin Li, Xianming Mo, Bo Xiang, Jing Chen

**Affiliations:** ^1^ Department of Pediatric Surgery and Laboratory of Pediatric Surgery, West China Hospital/West China School of Medicine, Sichuan University, Chengdu, China; ^2^ Laboratory of Molecular Developmental Biology, State Key Laboratory of Membrane Biology, Tsinghua-Peking Center for Life Sciences, School of Life Sciences, Tsinghua University, Beijing, China; ^3^ Department of Pediatric Surgery and Laboratory of Stem Cell Biology, State Key Laboratory of Biotherapy, West China Hospital, Sichuan University, Chengdu, China

**Keywords:** zebrafish, oocyte, Cut&Tag, histone modifications, epigentic

## Abstract

Histone modification signatures mark sites of transcriptional regulatory elements and regions of gene activation and repression. These sites vary among cell types and undergo dynamic changes during development and in diseases. Oocytes produce numerous maternal factors essential for early embryonic development, which are significantly influenced by epigenetic modifications. The profiling of epigenetic modifications during oogenesis remains uniquely challenging due to the presence of numerous tightly wrapped granulosa cells. Here, we successfully established a low-input CUT&Tag (Cleavage Under Targets and Tagmentation) method tailored for zebrafish stage I oocytes. This advanced technique enables high-resolution profiling of histone modifications and DNA-binding proteins, critical for understanding chromatin dynamics in developing oocytes. In this study, we detailed the workflow for this technique, including the isolation of pure stage I oocytes without somatic cells, library construction and quality monitoring. Our results demonstrate the method’s efficacy by identifying distinct histone modification patterns and analyzing differentially expressed genes in oocytes with and without granulosa cells. We also successfully profiled divergent histone modifications in oocytes derived from wild-type and *huluwa* mutants. These advancements overcome technical challenges in epigenetic research on zebrafish oocytes and establish a solid foundation for exploring the epigenetic regulatory mechanisms of maternal contribution.

## Introduction

Epigenetics, including DNA methylation, histone modification, and non-coding RNA, regulate gene expression by altering chromosome state without changing DNA sequences (Bird, 2007; Goldberg et al., 2007). These epigenetic modifications can be influenced by both external and internal factors and may be inherited (Goldberg et al., 2007). Early embryonic development is initially coordinated by maternal factors that accumulate in the egg during oogenesis and are progressively replaced by factors newly expressed in embryos (Zhang et al., 2022). During gametogenesis, germ cells undergo extensive and orderly epigenetic reprogramming. In primordial germ cells (PGCs) of mice, global methylation levels rapidly decrease during their migration to the gonads and are re-established during oocyte growth (Sasaki and Matsui, 2008). Concurrently, histone modifications also undergo global changes. Upon entering the genital ridge, germ cells undergo a transient increase in H3K4 methylation and H3K9 acetylation, modifications associated with transcriptionally active chromatin (Seki et al., 2005). Establishing proper epigenetic modifications during oogenesis and early embryo development is crucial. The success rate of developing mature metaphase II (MII) oocytes from primordial germ cell-like cells (PGC-like cells) induced by embryonic stem cells (ESC) and induced pluripotent stem cells (iPSC) *in vitro* is significantly lower (0.9%, or 26 out of 2,753) compared to the success rate in superovulated mice (61.7%) (Hikabe et al., 2016). This discrepancy is partly attributed to histone modification changes in gene promoter regions driven by Polycomb proteins, leading to premature gene activation (Blackledge and Klose, 2021). Polycomb proteins typically catalyze mono-ubiquitination of histone H2A at lysine 119 (H2AK119ub1) and H3K27me3 (Blackledge and Klose, 2021). DNMT1 is a key enzyme that maintains DNA methylation homeostasis in oocytes and embryos. Mouse oocytes lacking this enzyme develop into offspring exhibiting a loss of methylation at specific imprinting loci and allele-specific expression. This ultimately results in the death of homozygous female offspring during the final third of pregnancy (Ge et al., 2015; Howell et al., 2001). These findings highlight the crucial importance of establishing proper chromatin state during oocyte growth to ensure normal embryo development.

Transcriptional regulatory elements and their activation or silencing signatures are marked by various chromatin features that differ between cell types and change during development and disease progression. Initially, specific chromatin marks were studied using classical ChIP (Chromatin Immunoprecipitation) assays (Rodriguez-Ubreva and Ballestar, 2014). Advances in readout technologies have significantly improved these studies, with ChIP combined with high-throughput sequencing (ChIP-seq) greatly enhancing scope and efficiency. However, ChIP-seq still faces notable challenges, including small sample sizes, low signal-to-noise ratios, and susceptibility to man-made operation factor (Ai et al., 2019; Schmidl et al., 2015). In response to these limitations, enzyme-tethering *in situ* methods have gained popularity as an alternative. Over the past 2 decades, several enzyme-tethering methods have been developed, including DamID (DNA adenine methyltransferase Identification), ChEC (Chromatin Endogenous Cleavage), and ChIC (Chromatin Immuno Cleavage). In DamID, the chromatin protein of interest is fused with *Escherichia coli* Dam methyltransferase to methylate GATC motifs near binding sites. This is followed by cleavage with a GATC-specific restriction enzyme for localization (van Steensel and Henikoff, 2000). In ChIC, micrococcal nuclease (MNase) is indirectly linked to the antibody through a protein A-MNase fusion protein and cleaves DNA in the presence of calcium ions, avoiding the need to construct recombinant proteins (Schmid et al., 2004). In 2017, Steven Henikoff’s team developed CUT&RUN (Cleavage Under Targets and Release Using Nuclease) technology based on ChIC (Skene and Henikoff, 2017). Unlike ChIP, CUT&RUN does not require formaldehyde fixation, thus avoiding false positives or antibody epitope masking due to cross-linking. Instead, cells are permeabilized with digitonin and incubated with the target antibody, then combined with protein A/G-MNase, which cleaves chromatin near the targeted binding sites. CUT&RUN is compatible with high-throughput applications, providing a higher signal-to-noise ratio and a significant reduction in sequencing requirements. The introduction of CUT&Tag technology in 2019 constitutes the latest advancement in genomic chromatin profiling (Kaya-Okur et al., 2019). In CUT&Tag, the protein A-Tn5 (pA-Tn5) transposase replaces MNase. Upon activation by magnesium ions, this system integrates mosaic end adapters into adjacent DNA, creating fragments that can be amplified for sequencing libraries. The high integration efficiency of Tn5 transposase allows for high sensitivity, further reducing sample and sequencing requirements.

The dynamic changes in epigenetic modifications during oocyte development and the maternal-zygotic transition, which are closely linked to chromatin structure and histone modifications, have been extensively documented in mice. In contrast, although the zebrafish model has significantly contributed to other fields, few studies have taken advantage of this powerful model to study epigenetic modifications in oocytes. The main challenges hindering the application of the zebrafish oocytes as epigenetic model include limitations in understanding zebrafish ovaries, difficulties in obtaining oocytes, and the shortcomings of existing methods for analyzing chromatin accessibility and epigenetic modifications, particularly in specific cell types such as oocytes.

Zebrafish oocytes are surrounded by a layer of granulosa cells (GCs), which support the developing oocyte within the ovarian follicle. This creates a challenge for complete separation due to the large number and small volume of granulosa cells compared to oocytes (hundreds of granulosa cells surrounding a single oocyte). Even minimal contamination with a single granulosa cell can interfere with downstream analyses. Thus, removing granulosa cells is crucial for studies focusing on genomic and epigenetic characteristics. In previous research, we developed a method for the rapid and efficient isolation of stage I zebrafish oocytes free from granulosa cells. Oocytes are larger than other cells, and their chromatin is generally more loosely packed. However, after granulosa cell removal, we found that stage I oocytes were fragile and prone to crushing during repeated wash steps of the CUT&Tag procedure. Additionally, under standard fragmentation procedures, their chromatin was frequently over-digested by Tn5, leading to non-specific peaks.

To address these issues, we modified the CUT&Tag procedure based on our oocyte isolation method and successfully adapted CUT&Tag technology for zebrafish oocytes. This adapted method allows for the analysis of as few as 1,000 oocytes to obtain specific peaks for histone modifications. This advancement fills a gap in epigenetic research on zebrafish oocytes and provides a solid foundation for further exploring epigenetic regulatory mechanisms during oogenesis, with potential applications to other fish species.

## Results

### Rapid isolation of stage I zebrafish oocytes devoid of granulosa cells

**FIGURE 1:**
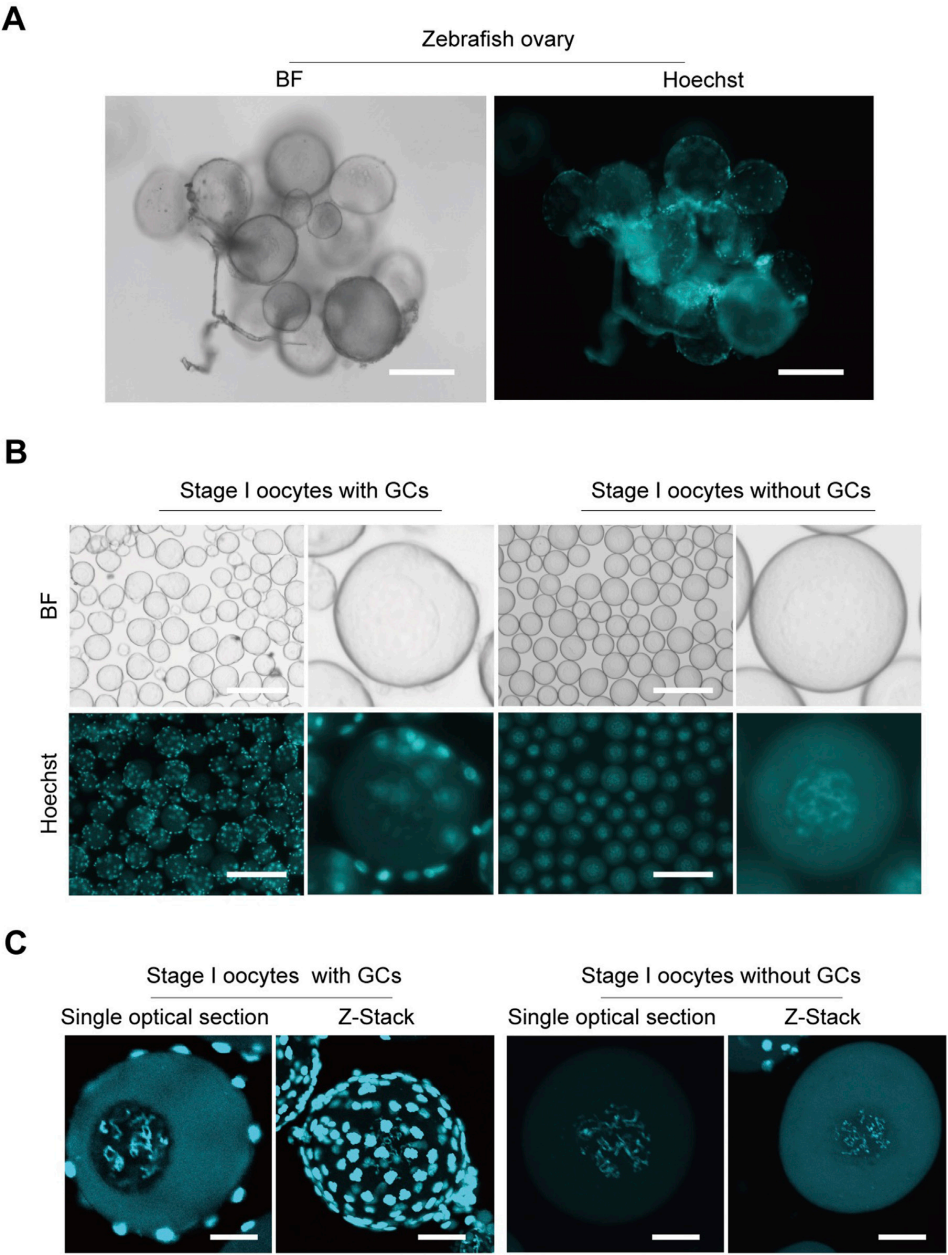
Isolation zebrafish stage I oocytes without granulosa cells. **(A)** Morphological imaging of zebrafish ovaries. **(B)** Isolated stage I oocytes with or without granulosa cells. **(C)** Z-Stack and single optical section images of oocytes with or without granulosa cells captured by confocal microscopy. BF, bright field; GCs, granulosa cells. Z-Stack, *Z*-axis projection by stacking; Scale bar: 100 μm **(A)**, 200 μm **(B)**, 20 μm **(C)**.

To ensure that oocytes for CUT&Tag are not confounded by granulosa cell interference, we developed a method to isolate pure stage I oocytes while eliminating granulosa cell contamination. Oocytes stained with Hoechst within the ovaries show small, bright granulosa cell nuclei tightly surrounding the oocytes (Figure 1A). Initially isolated stage I zebrafish oocytes still visibly contained granulosa cells, as observed by fluorescence microscopy. Through method refinement (Zheng et al., 2024), we achieved the isolation of stage I oocytes completely free of granulosa cells, nearly 100% pure oocytes (Figure 1B). Additionally, oocytes with or without surrounding granulosa cells were examined under confocal microscopy, yielding both Z-stack and single optical section images (Figure 1C). The presence of granulosa cells significantly impacts genomic sequencing, as their abundance can obscure the true information from the oocytes. This advancement establishes a crucial foundation for subsequent genome-related analyses and research.

### Impact of granulosa cells on RNA-seq of oocytes

**FIGURE 2:**
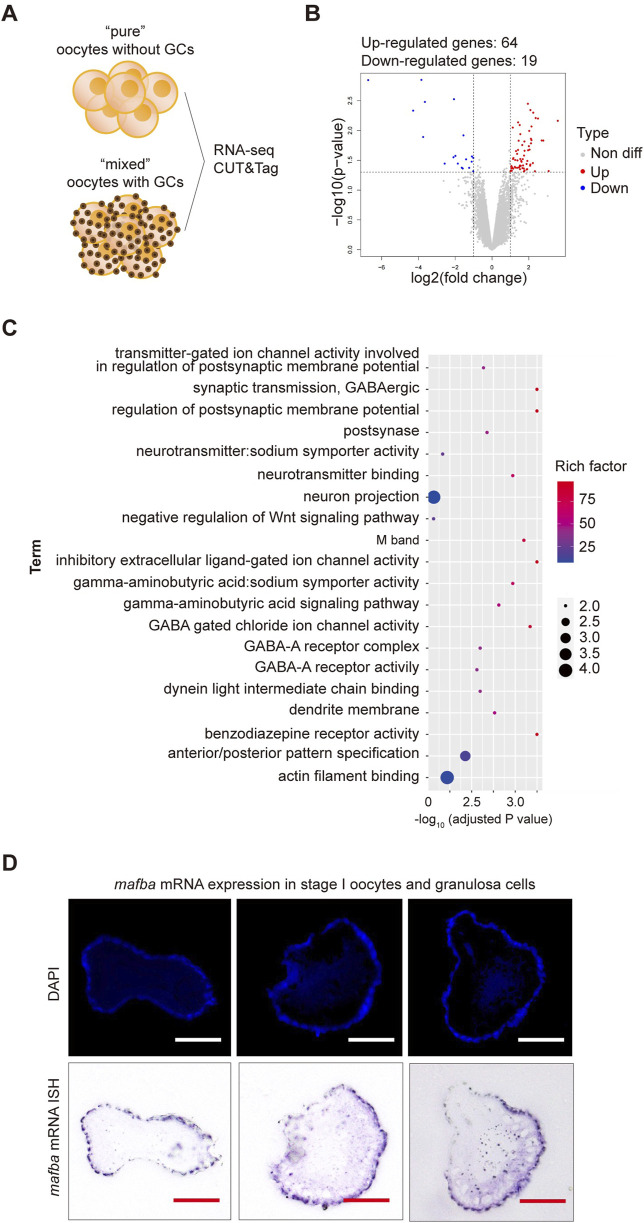
RNA sequencing analysis of differentially expressed genes (DEGs) between stage I oocytes with or without granulosa cells. **(A)** Schematic diagram of sequencing analysis. **(B)** Volcano plot of DEGs. “pure” sample versus “mixed” sample, upregulated and downregulated genes are indicated in red and blue, respectively. DEGs were identified based on the following criteria: *p*-value < 0.05 and log2|fold change| ≥ 1. **(C)** Gene Ontology (GO) enrichment analysis of differentially upregulated genes between stage I oocytes with or without granulosa cells. GO enrichment analysis was based on the following criteria: adjusted *p*-value < 0.05. **(D)** *In situ* hybridization (ISH) of *mafba* and DAPI staining of zebrafish ovary sections. Purple: RNA probe labeled *mafba* mRNA; Blue: DAPI labeled cell nuclei. Scale bar, 100 μm.

Previous findings suggest that the presence of granulosa cells in the cell pool does not significantly affect RNA sequencing results, as RNA sequencing shows high expression of germline-specific markers and meiotic genes, significant differential expression between oocyte stages, and low expression of follicle cell-specific genes (Elkouby and Mullins, 2017). To further test whether the inclusion of granulosa cells impacts RNA sequencing results, we conducted RNA sequencing on oocytes with and without granulosa cells (Figure 2A). Samples containing both oocytes and granulosa cells will be hereinafter referred to as “mixed,” whereas samples consisting solely of clean oocytes will be referred to as “pure”. Applying criteria (*p*-value <0.05 and log2|fold change| ≥ 1), only 83 differentially expressed genes (DEGs) were identified between two samples, including 64 upregulated and 19 downregulated genes, respectively (Figure 2B). Gene Ontology (GO) enrichment analysis (with adjusted *p*-value <0.05) of the differentially expressed genes revealed functional distinctions among a limited number of genes (Figure 2C). To validate our findings, *in situ* hybridization (ISH) analyses were conducted to examine the expression of *mafba*, one of the identified DEGs. Consistent with our RNA-Seq analyses, our ISH staining results confirmed that *mafba* was not expressed in stage I oocytes but was highly expressed in granulosa cells (Figure 2D). Besides, expression of *aldh1a2* (downregulated genes in pure oocytes) was previously reported to be detected in somatic cells, but not germ cells, in zebrafish female gonads throughout development (Rodríguez-Marí et al., 2013). What’s more, in GO enrichment analysis, *mafba* and *aldh1a2* are grouped to the same biological process, GO:0009952: anterior/posterior pattern specification, indicating their function in Retinoic acid signaling for follicle development. These findings indicate that although the interference of granulosa cells in RNA sequencing of oocytes is minimal (only 83 DEGs), due to relatively small volume and low RNA content for granulosa cells, some granulosa cell-specific genes can still be identified. Therefore, isolating completely clean and pure oocytes would greatly improve the accuracy of RNA sequencing results.

### Establishment of the CUT&Tag workflow for zebrafish oocytes

**FIGURE 3:**
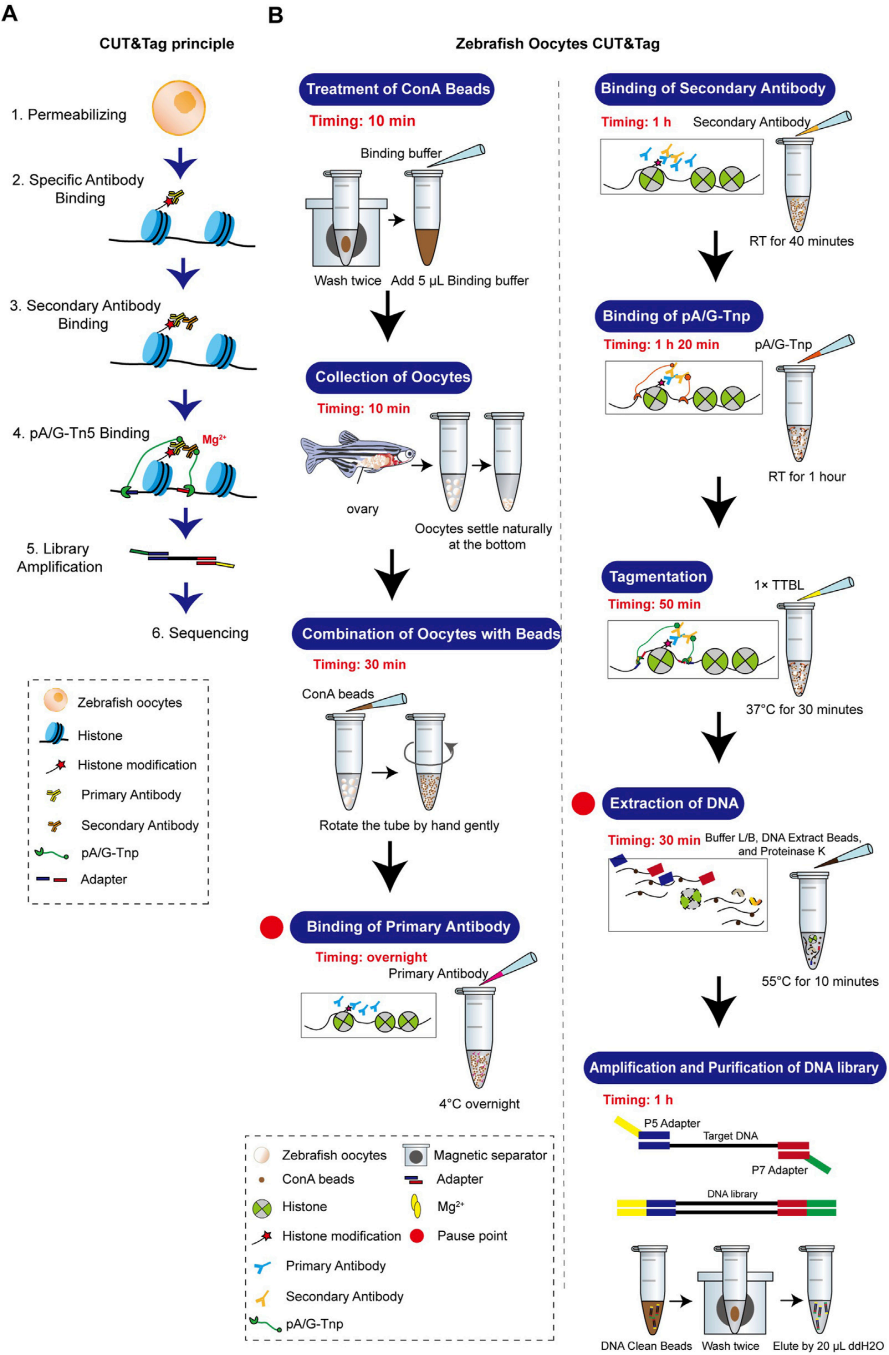
Zebrafish oocyte CUT&Tag principle and workflow. **(A)** Schematic diagram of CUT&Tag. **(B)** Zebrafish oocyte CUT&Tag workflow.

The standard CUT&Tag workflow was optimized to accommodate the unique characteristics of zebrafish oocytes. Our method achieves stable results with 1,000–3,000 oocytes. Details on the equipment, materials, and procedures are provided in the Supplementary Material, and a comprehensive flowchart is illustrated in Figure 3. Zebrafish oocytes are large and differ significantly in morphology and biochemical characteristics compared to somatic cells. They are also challenging to obtain in sufficient quantities and are prone to breakage after isolation. Therefore, minimizing sample loss throughout the entire process is crucial. To maximize collection efficiency during the Oocyte Collection step, natural sedimentation is recommended. The time for combining ConA beads and oocytes should be increased, while the Tagmentation time should be reduced. All steps prior to DNA extraction should avoid centrifugation and vigorous pipetting as much as possible. Additionally, the CUT&Tag procedure for oocytes is temperature-sensitive. Thus, the room temperature during this method should be maintained at 25°C.

### Quality control for oocyte CUT&Tag

**FIGURE 4:**
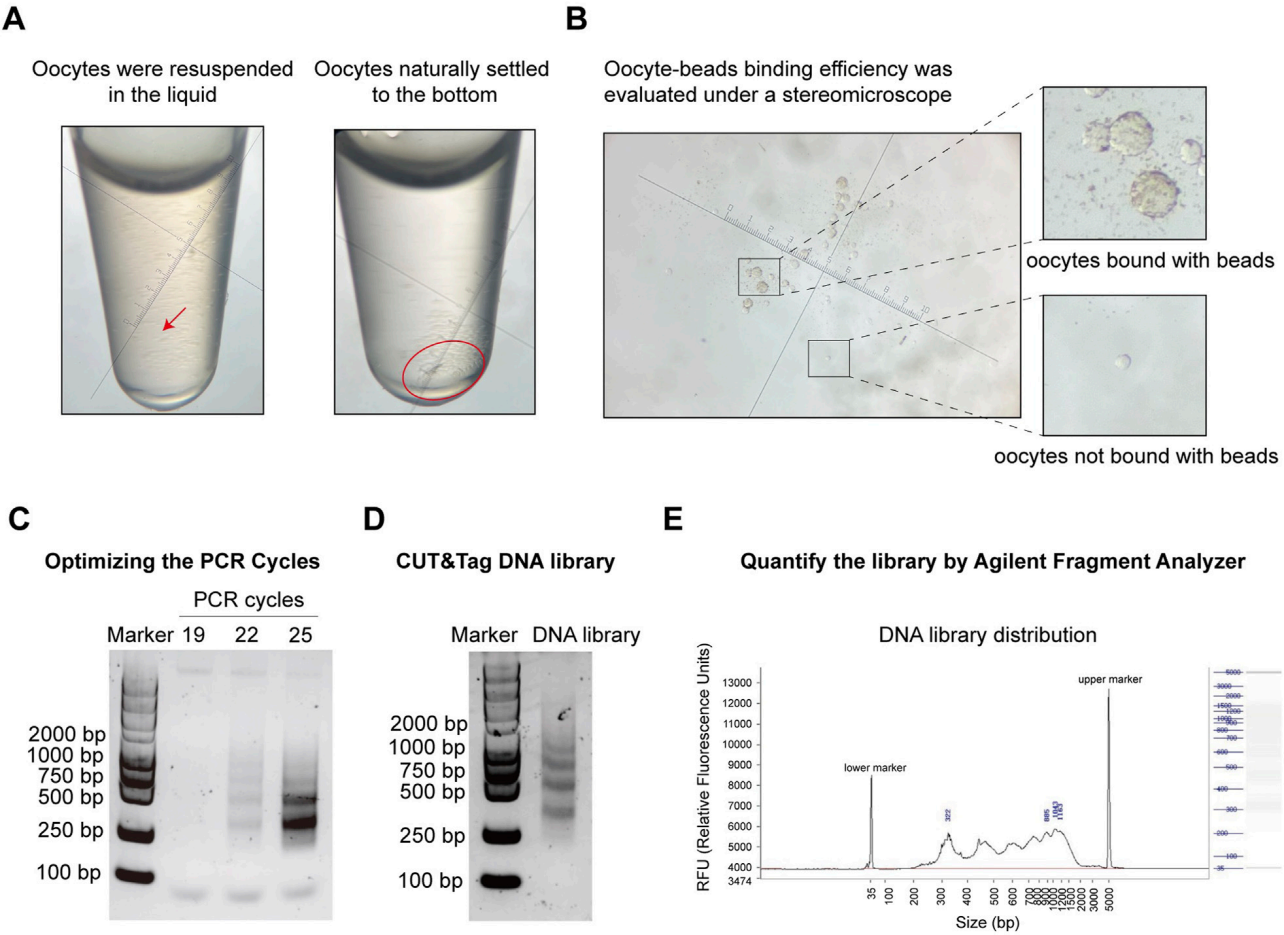
Pre-sequencing quality control of oocyte CUT&Tag. **(A)** Images of oocyte collection in a tube under a stereomicroscope. Left panel: oocytes resuspended in liquid; Right panel: oocytes settled at the bottom by gravity. Magnification = 45x. **(B)** Images of ConA beads-oocyte complex and unbound oocytes under a stereomicroscope. Magnification = 45x. **(C)** Agarose gel electrophoresis images for PCR cycles test with different PCR cycles. **(D)** Agarose gel electrophoresis images of the final CUT&Tag library. **(E)** Electrophoresis of the final DNA library and the distribution analysis using the DNF-915 Fragment Analyzer.

Several quality control steps are essential to achieve optimal results in the oocyte CUT&Tag procedure. Due to the fragility of oocytes, centrifugation and pipetting should be avoided during oocyte collection and washing (steps 2.4–2.7). Instead, cells should be gently resuspended by hand rotation and allowed to settle naturally for 1–2 min. The collection tube should be observed under a stereomicroscope, and once all cells have settled at the bottom, the supernatant should be carefully removed (Figure 4A). When combining the oocytes with ConA beads, the supernatant after binding should be observed under a stereomicroscope to assess binding efficiency and collection efficiency (Figure 4B). The number of PCR cycles directly impacts the quality of the DNA library. Insufficient cycles may result in incomplete amplification of target DNA fragments, while excessive cycles can lead to over-enrichment of biased DNA fragments. Prior to library amplification, a preliminary PCR must be conducted to determine the optimal number of cycles. For 1,000–3,000 cells, initial testing was performed with 19, 22, and 25 cycles for pre-amplification. Depending on the cell count and the abundance of target proteins, multiple cycle conditions should be tested in the pre-amplification to establish the optimal range. Agarose gel electrophoresis images from steps 2.34–2.37 indicate results for three candidate cycle numbers (Figure 4C). A visible DNA band was observed at 22 cycles, leading to the selection of 18 cycles as the final PCR cycle number (Figure 4D). Final DNA libraries were checked using electrophoresis and the DNF-915 Fragment Analyzer. A high-quality histone library is characterized by nucleosome periodicity with DNA fragment sizes ranging from 200 bp to 1,000 bp, serving as preliminary quality control before sequencing (Figure 4E).

### Granulosa cells greatly influence the epigenetic analysis of oocytes

To explore the potential impact of granulosa cell contamination on genome-related studies, CUT&Tag analysis was employed to investigate the modifications of H3K4me3, H3K4me1, H3K27ac, and H3K27me3 in GCs-mixed and pure oocytes. These histone modifications serve as critical signals for transcriptional activation or repression. Specifically, H3K4me3, H3K4me1, and H3K27ac are typically associated with transcriptional activation: H3K4me1 and H3K27ac for active enhancers and H3K4me3 and H3K27ac for active promoters. Meanwhile, H3K27me3 modification is associated with the downregulation of nearby genes through recruiting repressive factors, (Ferrari et al., 2014; Lawrence et al., 2016).

**FIGURE 5:**
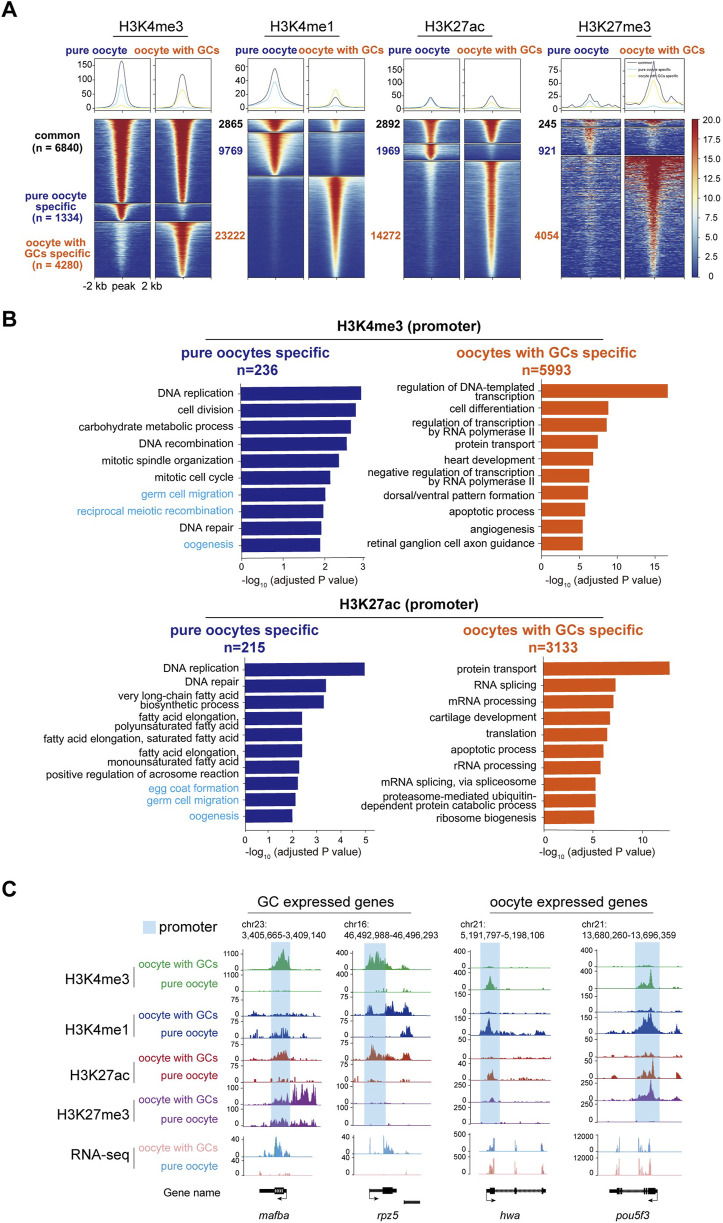
CUT&Tag analysis of histone modifications between stage I oocytes with or without granulosa cells. **(A)** Heatmap showing the H3K4me3, H3K4me1, H3K27ac, and H3K27me3 enrichment around peak center (peak center ±2 kb) in pure oocytes and oocytes with GCs samples. Peaks were classified into three clusters based on their dynamics between samples: common (present in both samples), pure oocyte-specific (only present in pure oocytes sample), and oocyte with GCs-specific (only present in the mixed sample). The numbers of peaks in each group were labeled. **(B)** Barplots showing the top 10 GO terms enriched for genes whose TSS were located within ±2 kb of H3K4me3 (upper) or H3K27ac (lower) peaks specific in pure oocytes (left, blue) or mixed samples (right, orange). Oogeneisis and germ cell related terms were highlighted in light blue. **(C)** H3K4me3, H3K4me1, H3K27ac, and H3K27me3 modifications near differentially GC expressed genes (*mafba* and *rpz5*) and oocyte expressed maternal genes (*hwa* and *pou5fa*) in stage I oocytes with or without granulosa cells. Promoter of each gene was labeled in light blue. GCs, granulosa cells.

Heat map analysis of the H3K4me3, H3K4me1, H3K27ac, and H3K27me3 enrichment around peak center (peak center ±2 kb) identified differentially enriched peaks in pure oocytes and oocytes with GCs samples. Peaks were classified into three clusters based on their dynamics: common (present in both samples), oocyte-specific (only present in pure oocytes sample), and oocyte with GCs-specific (only present in the mixed sample), with the numbers of peaks (n) in each group (Figure 5A). As H3K4me3 and H3K27ac are usually associated with transcriptional active promoters. Gene Ontology (GO) enrichment analysis (with adjusted *p*-value <0.05) of the differentially enriched genes, whose TSS were located within ±2 kb of H3K4me3 or H3K27ac, revealed functional distinctions among two samples (Figure 5B). Interestingly, Germ cell and oogenesis related GO terms (highlight in light blue in Figure 5B) were enriched in pure oocytes-specific peaks, but not in the mixed samples. This strengthens the importance of oocyte purity for oocyte transcriptional activity analysis.

Our results revealed striking differences in these four histone modifications across the genome in both oocyte groups. To further investigate the changes in these modifications near specific gene loci and their relationship with transcription, we first focused on the *mafba* locus, a differentially expressed transcription factor in the mixed oocyte sample (containing oocytes and granulosa cells) identified by RNA-seq analysis. Abundant modifications of H3K4me3 and H3K27ac were found at the promoter of *mafba* gene in the mixed oocyte sample, while absent in the pure oocytes (Figure 5C). These active modifications align with the transcriptional activity of *mafba* in granulosa cells. Similar patterns were observed for other granulosa cell-specific genes, such as *rpz5* (Figure 5C).

On the other hand, we examined histone modifications near some important maternal genes, which are typically transcribed in oocytes and deposited in eggs before fertilization (Barckmann and Simonelig, 2013). One of these important maternal genes is *huluwa (hwa)*, which functions as a dorsal determinant to dictate body axis formation in vertebrates (Yan et al., 2018). CUT&Tag analysis revealed the presence of active histone modifications, such as H3K4me1, H3K4me3, and H3K27ac, at the *hwa* locus in the pure oocytes. Conversely, repressive H3K27me3 modification was more abundant in the mixed oocyte sample (Figure 5C), aligning with the expression pattern of *hwa* mRNA. A similar modification pattern was observed for *pou5f3* (Figure 5C).

Besides, we also checked histone modifications in well-known granulosa cells-specific genes. Abundant H3K4me1, H3K4me3, and H3K27ac modifications were observed near the gene loci of *gsdf* and *amh* in the mixed oocyte sample, whereas these modifications were absent in the pure oocyte sample. These two genes have been reported to be specifically expressed in gonad somatic cells (Gautier et al., 2011; Rodríguez-Marí et al., 2005), even though they were not identified as DEGs in our RNA-seq. That is because the actual differences in RNA expression may be masked by the overall differences in expression levels, for the “mixed” sample (granulosa cells and oocytes) is a mixture of 2 cell types, with big difference in volume and RNA content.

Our comprehensive CUT&Tag analyses demonstrated that granulosa cell contamination could significantly disturb the profiling of histone modifications of oocytes, which might be the obstacle for regulatory element identification during oogenesis. These findings highlight the importance of isolating pure oocytes to ensure the reliability of genome- and epigenome-related studies.

### CUT&Tag unveiled distinct histone modifications at *hwa* locus between WT and the *hwa* expression-deficient mutant oocytes

**FIGURE 6:**
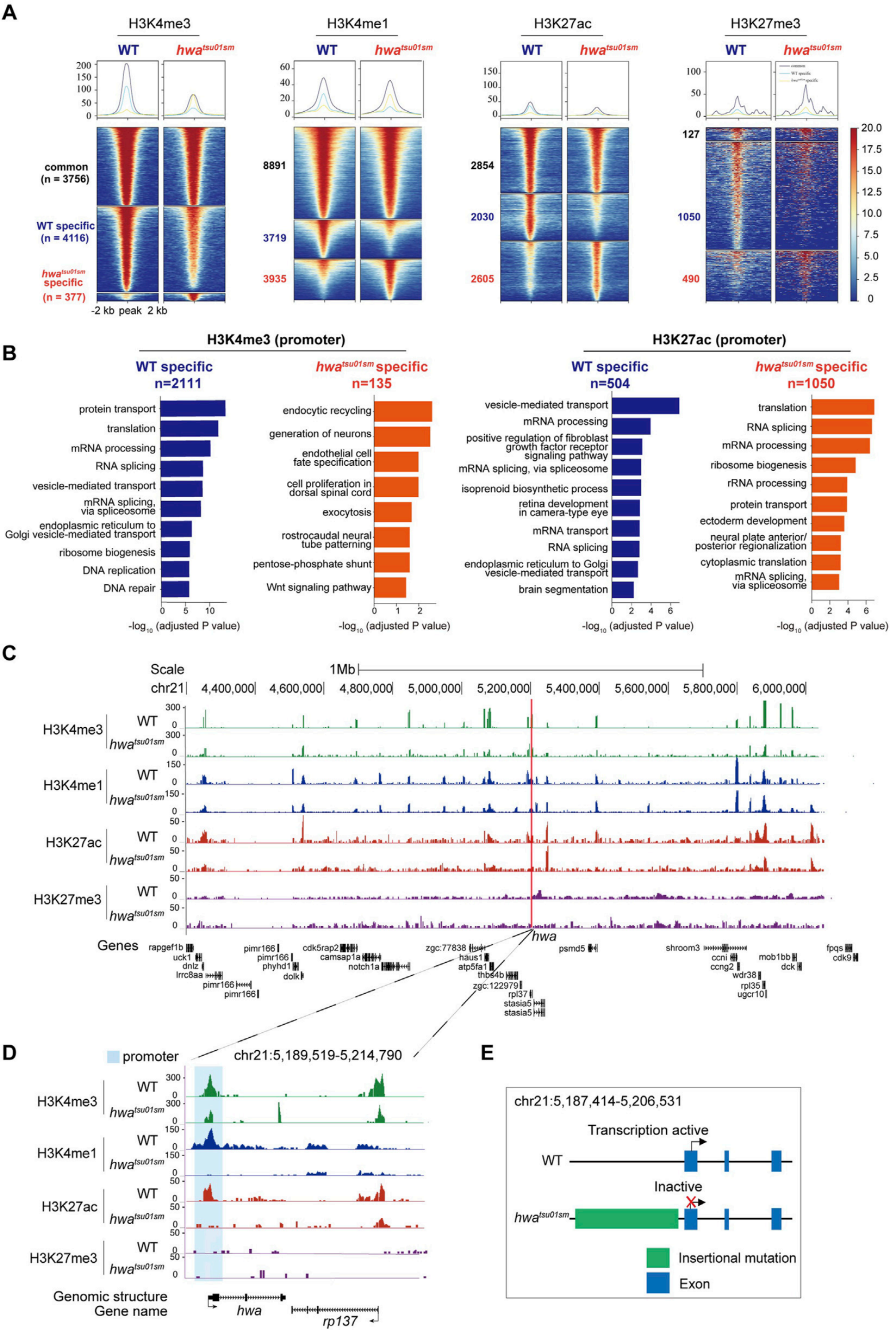
Distinct histone modifications around *hwa* locus between WT and *hwa* ^*tsu01sm*^ mutants. **(A)** Heatmap showing the H3K4me3, H3K4me1, H3K27ac, and H3K27me3 enrichment around peak center (peak center ±2 kb) in WT and *hwa* ^tsu01sm^ mutant pure oocytes. Peaks were classified into three clusters based on their dynamics between two samples: common (present in both WT and mutant samples), WT-specific (only present in WT oocytes), and *hwa*-specific (only present in mutant oocytes). The numbers of peaks in each group were also labeled. **(B)** Barplots showing the top 10 GO terms enriched for genes whose TSS were located within ±2 kb of H3K4me3 (left) or H3K27ac (right) peaks specific in WT oocytes (blue) or mutant oocytes (orange). **(C)** H3K4me3, H3K4me1, H3K27ac and H3K27me3 modifications landscapes at a region including 1 Mb upstream and downstream of *hwa* locus in the WT and *hwa* ^*tsu01sm*^ oocytes. **(D)** H3K4me3, H3K4me1, H3K27ac, and H3K27me3 modifications at the *hwa* locus in the WT and *hwa* ^*tsu01sm*^ oocytes. **(E)** Schematic diagram of gene structure and expression activity of WT and *hwa* ^*tsu01sm*^ mutant. WT, wild type; *hwa* ^*tsu01sm*^, *hwa* expression-deficient mutant with a retrotransposon insertion.

With this verified CUT&Tag method, we could also set out to address how a specific gene is turned on or off during oogenesis. *Hwa* is a recently identified maternal gene essential for dorsoventral axis formation, which is specifically expressed in oocytes and eggs and rapidly degraded after zygote genome activation (∼3 h post-fertilization). In a *hwa* expression-deficient mutant (*hwa* ^*tsu01sm*^) zebrafish model, a retrotransposon insertion results in transcriptional inactivation (Yan et al., 2018). However, the mechanisms underlying this insertional mutation remained elusive and warrant further investigation. Heat map analysis of the H3K4me3, H3K4me1, H3K27ac, and H3K27me3 enrichment around peak center (peak center ±2 kb) identified differentially enriched peaks in WT and *hwa* ^*tsu01sm*^ mutant oocytes. Peaks were classified into three clusters based on their dynamics: common (present in both samples), WT-specific (only present in WT oocytes), and *hwa* ^*tsu01sm*^ mutant-specific (only present in mutant oocytes) (Figure 6A). What’s more, Gene Ontology (GO) enrichment analysis (with adjusted *p*-value <0.05) of the differentially enriched genes, whose TSS were located within ±2 kb of H3K4me3 or H3K27ac peaks, revealed functional distinctions among these two samples (Figure 6B). These differential enrichments provide further opportunity to uncover the function and mechanism of *hwa* gene on oocyte development, which is not fully understood yet. Given that Hwa is a transmembrane protein functioning by activating β-catenin, genes enriched in GO terms, such as protein transport, ER-Golgi transport, endocytic recycling, exocytosis, vesicle-mediated transport, Wnt signaling pathway, need further investigation.

Another important question is how *hwa* gene is specifically activated during oogenesis. These histone modifications provided indicative information on the regulatory landscape of *hwa* gene within a region flanking 1 Mb upstream and downstream, which is supposed to be pivotal for transcription regulation. Notably, this region was rich in a plethora of specific peaks that align with the nearby genes, but coordinated differential histone modification peaks (H3K4me1and H3K27ac and H3K4me3) mainly located around the *hwa* locus (Figure 6C). Specifically, at *hwa* gene locus, H3K4me1 and H3K27ac modifications were absent and H3K4me3 modifications decreased at the promoter region in *hwa* ^*tsu01sm*^ mutant oocytes (Figure 6D). These changes in histone modifications in *hwa* ^*tsu01sm*^ mutant is consistent with the transcription activity (Figure 6E). The absence of H3K4me1 may suggest a reduction in enhancer activity, leading to decreased transcriptional initiation and gene expression. The absence of H3K27ac and the decrease in H3K4me3 indicate that the *hwa* promoter in mutant oocyte is inactive, preventing the recruitment of the transcriptional machinery necessary for gene expression.

The inactivation of the *hwa* gene in *hwa* ^*tsu01sm*^ due to a retrotransposon insertion mutation, along with the loss of active histone marks, suggests that these modifications are essential for maintaining gene expression. Further investigations examining other histone modifications and assessing changes in chromatin accessibility could elucidate how the chromatin landscape at the *hwa* locus is altered in *hwa* ^*tsu01sm*^ mutant oocytes and their impact on maternal gene expression. These findings support the immense potential of the oocyte CUT&Tag technique in addressing the mechanism of transcriptional regulation of specific genes.

## Discussion

In this study, the CUT&Tag technology was optimized for use with zebrafish oocytes in small numbers, demonstrating that as few as 1,000 oocytes can yield reliable and specific peaks for histone modifications. The dynamic changes in epigenetic modifications during oocyte development and the maternal-zygotic transition are closely linked to chromatin state and histone modifications. The optimized CUT&Tag method enables detailed mapping of these modifications, providing crucial insights into the epigenetic landscape of oocytes. This capability is particularly important for understanding the mechanisms underlying maternal gene regulation and the transition to zygotic gene expression. Our findings also emphasize the critical importance of eliminating granulosa cells from oocyte samples to ensure accurate genetic and epigenetic analyses. Significant differences were observed in histone modification profiles generated from oocyte samples with or without granulosa cell contamination. Granulosa cell contamination can significantly influence the interpretation of histone modification data, potentially leading to incorrect conclusions about gene regulation in oocytes.

It is noteworthy that there are more peaks for all the tested histone modifications in the mixed samples than in the pure ones (Figure 5A). We suppose this may stem from biological difference of the samples. When we carried out the CUT&Tag experiment, we used the same/comparable number of oocytes (about 1,000). For the mixed samples, there have more cells actually (considering hundreds of granulosa cells wrap around a single oocyte). To get a more reliable result, the mapped reads used for downstream peak calling and analysis between mixed and pure oocytes were comparable. Although the volume and RNA content of granulosa cell is very low, the nuclear contribution/contamination is obvious. We think the massive CUT&Tag peaks in mixed samples were from the granulosa cells’ nuclei. Besides, most of the peaks generated in mixed samples are not oogenesis/germ cell related, but more general biological aspects, e.g., protein transport, RNA processing, apoptotic process instead. Furthermore, the CUT&Tag of WT and *hwa* ^tsu01sm^ mutant pure oocytes generated comparable number of peaks, indicating the CUT&Tag method we established is reproducible and reliable. Thus, the massive contribution of Cut&Tag peaks from granulosa cells further strengthens our claim that pure oocyte isolation is extremely important for nucleus/genome based analysis.

Gene expression in oocytes and somatic cells is extensively regulated by alterations in epigenetic modifications. The H3K4me3 modification enhances transcription factor accessibility to DNA by reshaping chromatin, thereby facilitating gene transcription and expression (Howe et al., 2017; Wang et al., 2023). H3K27ac is typically found in the proximal and distal regions of the transcription start site (TSS) and is defined as a marker for active promoters and enhancers, respectively (Heintzman et al., 2009; Heintzman et al., 2007; Howe et al., 2017; Wang et al., 2023). In this study, we observed distinct modifications near genes specifically expressed in granulosa cells, such as *mafba*, *rpz5*, and *gsdf*, with H3K4me3 and H3K27ac modification significantly enriched at their promoters in mixed samples. For the maternally expressed genes *hwa* and *pou5f3*, H3K4me3 and H3K27ac were significantly enriched in pure oocytes. However, in granulosa cell-dominant genes, these active modifications were no longer present and were instead replaced by the repressive marker H3K27me3. This indicates the potential role of histone modifications in the regulation of gene expression during germ cell development and vividly demonstrates the dynamic changes of histone modifications with cell fate specification. Most previous studies have focused on the zygotic stage post-fertilization, whereas the oogenesis process in fish has remained largely unexplored. This optimized methodology could enable related studies and will benefit other researchers.

In conclusion, the optimized CUT&Tag method for zebrafish oocytes represents a significant advancement in maternal regulation research. It provides a robust and sensitive tool for investigating histone modifications and other epigenetic changes in oocytes, addressing previous technical limitations. This method will contribute to understanding the epigenetic mechanisms governing oogenesis and early embryonic development.

## Materials and methods

### Zebrafish

The wild-type AB strain and *hwa* ^*tsu01sm*^ zebrafish raised at 28.5C with a 14 h:10 h light and dark cycle were used in this study. Maintenance and handling of zebrafish has been approved by local authorities and by the animal ethics committee of the West China Hospital of Sichuan University (approval number 20220422003). All operation of euthanasia follow international animal welfare guidelines (AVMA, 2020; OLAW, 2015).

### Regents and equipments

The regents used in this research include Leibovitz’s L-15 medium, with L-glutamine (Hyclone, SH30525.01), H3K27Ac antibody (Abcam, ab4729), H3K4me3 antibody (Active motif, 39,060), H3K27me3 antibody (Diagenode, C10410069), Cell Dissociation Solution (Kinger’s solution, PC-33689), Hoechst (Yesen, 40732ES03), Hyperactive Universal CUT&Tag Assay Kit for Illumina (Vazyme, TD903), TruePrep Index Kit V2 for Illumina (Vazyme, TD202), and equipments include 100 μm cell strainers (Falcon, 352,360), Forceps (Dumont #5), Vannas spring scissors (FTS #15000–00), Stereomicroscope (Motic, SMZ-161), 6-well Tissue Culture Plate (SORFA, 220,100), 35 mm Plastic dish (SORFA, 230,301), 10 μL low adsorption pipette tips (Labsellect, T-0010-LR-R-S), 28.5°C incubator (WIGGENS, WH-01), Fluorescence microscope (Zeiss, Axio Zoom. V16), glass capillary needle that blunted by burning with lighter.

### Isolation of stage I oocytes of zebrafish without granulosa cells

Female zebrafish with the standard length (SL) ranging from 10 mm to 15 mm were chosen to dissect the ovaries. Rapid chilling (hypothermic shock) method was employed to euthanize the zebrafish by placing the juvenile female fish in ice-cold water (Ferreira et al., 2022; Matthews and Varga, 2012). To ensure euthanasia and minimize pain experienced by zebrafish, the following steps were used: make an ice-chilled water bath (0°C–4°C), swiftly transfer females to the ice-cold water, and subsequent hold fish in ice-chilled water for at least 10 min after loss of orientation and operculum movements. Cut off the head and tail by micro-scissors. Transfer the middle trunk region to a 35 mm dish containing 2 mL of L-15 medium (with L-glutamine) and dissect the ovaries. Transfer the ovaries to a 6-well plate containing 2 mL Cell Dissection Solution and incubate for 2–3 h at 28.5°C. Put a 100 μm cell strainer into the other well of 6-well plate, add L-15 medium, and make the medium level higher than the filter. Draw the digestive medium through the cell strainer using a pipette. Waiting 1–2 min until all the stage I oocytes have passed through the strainer then remove excess medium. Wash the stage I oocytes 5–6 times until there are no other impurities. Under a microscope with a magnification equal to or exceeding 10x, remove cell fragments, other-stage oocytes, and some stage I oocytes that are adhered by granulosa cells using a tool that can be manipulated with precision, such as a blunt injection needle. A final concentration of 5 μg/mL Hoechst 33,342 can be added to the L-15 medium to pick out the oocytes that do not meet the desired criteria.

### *In situ* hybridization of zebrafish ovaries

The ovaries were fixed in 4% paraformaldehyde for 24 h. Dehydration and rehydration are performed using a methanol gradient. Ovaries are treated with a prehybridization solution at 65°C for 4 h. The digoxygenin labeled RNA probes is applied to hybridization at 65°C overnight. Washing with a series of solutions to remove unbound or nonspecifically bound probes. Anti-Dig antibody which specific binding to digoxygenin are introduced to bind to the Dig-labeled RNA probe. Secondary antibody, conjugated to alkaline phosphatase is added to bind to the primary anti-Dig antibody. The substrate solution containing alkaline phosphatase substrates (BCIP/NBT, 5-bromo-4-chloro-3-indolyl phosphate/nitro blue tetrazolium) is added to producing a visible color.

### Frozen section

The ovaries after ISH were embeded in OCT compound. Place a cryomold on dry ice to pre-chill. Fill the cryomold with OCT compound, and carefully embed the dissected ovaries in it. Freeze the embedded tissue by placing the cryomold on dry ice for rapid solidification. Adjust the cryostat temperature to the −20°C to −25°C. Section the embedded tissue into thin slices (typically 10 μm). Pick up the sections using a brush or forceps and transfer them onto pre-chilled glass slides. Allow the sections to adhere to the slides, ensuring minimal folding or distortion. Perform staining on the sections following standard protocols.

### Oocyte RNA-seq

2000 oocytes of mixed and pure sample were applied for total RNA extraction using TRIzol (Invitrogen). Genomic DNA were removed by DNaseI (Novoprotein). RNA quality was measured by examining A260/A280 with Nanodrop (Thermo Fisher Scientific Inc.) and quantified by Qubit3.0 with QubitTM RNA Broad Range Assay kit (Life Technologies). RNA sequencing and analysis were performed by SEQHEALTH Biotechnology Corporation. Differentially expressed genes (DEGs) were identified from the RNA-seq data using the edgeR package. *P*-value < 0.05 and log2|fold change| ≥1 were used to identify significantly differentially expressed genes (DEGs). GO enrichment analysis was based on the criteria of adjusted *p*-value < 0.05.

### CUT&Tag data processing

CUT&Tag library sequencing and analysis were performed by Novogene Biotechnology Corporation. All reads were aligned to the zebrafish reference genome (danRer11) using Bowtie2 (version 2.2.2) (Langmead and Salzberg, 2012) with the parameters–t –q–N 1 –L 25. All unmapped reads, nonuniquely mapped reads, and PCR duplicates were removed. To visualize the signals in the UCSC Genome Browser, each read was extended by 250 bp, and the coverage for each base was counted. Peaks were called using MACS2 (Zhang et al., 2008) with the parameters -p1e-4 --nomodel -g 1.3e10. The called peaks with weak signals were filtered in the further analysis. Znf706 peaks located within ±2 kb of annotated TSS (transcription start site) were identified as promoter peaks. Other peaks were classed into distal peaks. Genes within ±50 kb of distal peaks were identified as potential enhancer target genes.

## Data Availability

Publicly available datasets were analyzed in this study. This data can be found here: All related data are included in the manuscript or supplementary materials.

## References

[B1] Ai S. Xiong H. Li C. C. Luo Y. Shi Q. Liu Y. (2019). Profiling chromatin states using single-cell itChIP-seq. Nat. Cell Biol. 21, 1164–1172. 10.1038/s41556-019-0383-5 31481796

[B2] AVMA (2020). AVMA guidelines for the euthanasia of animals. 2020 Edition.

[B3] Barckmann B. Simonelig M. (2013). Control of maternal mRNA stability in germ cells and early embryos. Biochim. Biophys. Acta 1829, 714–724. 10.1016/j.bbagrm.2012.12.011 23298642

[B4] Bird A. (2007). Perceptions of epigenetics. Nature 447, 396–398. 10.1038/nature05913 17522671

[B5] Blackledge N. P. Klose R. J. (2021). The molecular principles of gene regulation by Polycomb repressive complexes. Nat. Rev. Mol. Cell Biol. 22, 815–833. 10.1038/s41580-021-00398-y 34400841 PMC7612013

[B6] Elkouby Y. M. Mullins M. C. (2017). Methods for the analysis of early oogenesis in Zebrafish. Dev. Biol. 430, 310–324. 10.1016/j.ydbio.2016.12.014 27988227 PMC5555829

[B7] Ferrari K. J. Scelfo A. Jammula S. Cuomo A. Barozzi I. Stutzer A. (2014). Polycomb-dependent H3K27me1 and H3K27me2 regulate active transcription and enhancer fidelity. Mol. Cell 53, 49–62. 10.1016/j.molcel.2013.10.030 24289921

[B8] Ferreira J. M. Felix L. Jorge S. Monteiro S. M. Olsson I. A. S. Valentim A. M. (2022). Anesthesia overdose versus rapid cooling for euthanasia of adult zebrafish. Zebrafish 19, 148–159. 10.1089/zeb.2022.0001 35759370

[B9] Gautier A. Le Gac F. Lareyre J. J. (2011). The gsdf gene locus harbors evolutionary conserved and clustered genes preferentially expressed in fish previtellogenic oocytes. Gene 472, 7–17. 10.1016/j.gene.2010.10.014 21047546

[B10] Ge Z. J. Schatten H. Zhang C. L. Sun Q. Y. (2015). Oocyte ageing and epigenetics. Reproduction 149, R103–R114. 10.1530/REP-14-0242 25391845 PMC4397590

[B11] Goldberg A. D. Allis C. D. Bernstein E. (2007). Epigenetics: a landscape takes shape. Cell 128, 635–638. 10.1016/j.cell.2007.02.006 17320500

[B12] Heintzman N. D. Hon G. C. Hawkins R. D. Kheradpour P. Stark A. Harp L. F. (2009). Histone modifications at human enhancers reflect global cell-type-specific gene expression. Nature 459, 108–112. 10.1038/nature07829 19295514 PMC2910248

[B13] Heintzman N. D. Stuart R. K. Hon G. Fu Y. Ching C. W. Hawkins R. D. (2007). Distinct and predictive chromatin signatures of transcriptional promoters and enhancers in the human genome. Nat. Genet. 39, 311–318. 10.1038/ng1966 17277777

[B14] Hikabe O. Hamazaki N. Nagamatsu G. Obata Y. Hirao Y. Hamada N. (2016). Reconstitution *in vitro* of the entire cycle of the mouse female germ line. Nature 539, 299–303. 10.1038/nature20104 27750280

[B15] Howe F. S. Fischl H. Murray S. C. Mellor J. (2017). Is H3K4me3 instructive for transcription activation? Bioessays 39, 1–12. 10.1002/bies.201600095 28004446

[B16] Howell C. Y. Bestor T. H. Ding F. Latham K. E. Mertineit C. Trasler J. M. (2001). Genomic imprinting disrupted by a maternal effect mutation in the Dnmt1 gene. Cell 104, 829–838. 10.1016/s0092-8674(01)00280-x 11290321

[B17] Kaya-Okur H. S. Wu S. J. Codomo C. A. Pledger E. S. Bryson T. D. Henikoff J. G. (2019). CUT&Tag for efficient epigenomic profiling of small samples and single cells. Nat. Commun. 10, 1930. 10.1038/s41467-019-09982-5 31036827 PMC6488672

[B18] Langmead B. Salzberg S. L. (2012). Fast gapped-read alignment with Bowtie 2. Nat. Methods 9, 357–359. 10.1038/nmeth.1923 22388286 PMC3322381

[B19] Lawrence M. Daujat S. Schneider R. (2016). Lateral thinking: how histone modifications regulate gene expression. Trends Genet. 32, 42–56. 10.1016/j.tig.2015.10.007 26704082

[B20] Matthews M. Varga Z. M. (2012). Anesthesia and euthanasia in zebrafish. ILAR J. 53, 192–204. 10.1093/ilar.53.2.192 23382350

[B21] OLAW (2015). PHS policy on humane care and use of laboratory animals. 10.1080/10888700903579838 20349378

[B22] Rodríguez-Marí A. Cañestro C. BreMiller R. A. Catchen J. M. Yan Y. L. Postlethwait J. H. (2013). Retinoic acid metabolic genes, meiosis, and gonadal sex differentiation in zebrafish. PLoS One 8, e73951. 10.1371/journal.pone.0073951 24040125 PMC3769385

[B23] Rodríguez-Marí A. Yan Y. L. Bremiller R. A. Wilson C. Cañestro C. Postlethwait J. H. (2005). Characterization and expression pattern of zebrafish Anti-Müllerian hormone (Amh) relative to sox9a, sox9b, and cyp19a1a, during gonad development. Gene Expr. Patterns 5, 655–667. 10.1016/j.modgep.2005.02.008 15939378

[B24] Rodriguez-Ubreva J. Ballestar E. (2014). Chromatin immunoprecipitation. Methods Mol. Biol. 1094, 309–318. 10.1007/978-1-62703-706-8_24 24162998

[B25] Sasaki H. Matsui Y. (2008). Epigenetic events in mammalian germ-cell development: reprogramming and beyond. Nat. Rev. Genet. 9, 129–140. 10.1038/nrg2295 18197165

[B26] Schmid M. Durussel T. Laemmli U. K. (2004). ChIC and ChEC; genomic mapping of chromatin proteins. Mol. Cell 16, 147–157. 10.1016/j.molcel.2004.09.007 15469830

[B27] Schmidl C. Rendeiro A. F. Sheffield N. C. Bock C. (2015). ChIPmentation: fast, robust, low-input ChIP-seq for histones and transcription factors. Nat. Methods 12, 963–965. 10.1038/nmeth.3542 26280331 PMC4589892

[B28] Seki Y. Hayashi K. Itoh K. Mizugaki M. Saitou M. Matsui Y. (2005). Extensive and orderly reprogramming of genome-wide chromatin modifications associated with specification and early development of germ cells in mice. Dev. Biol. 278, 440–458. 10.1016/j.ydbio.2004.11.025 15680362

[B29] Skene P. J. Henikoff S. (2017). An efficient targeted nuclease strategy for high-resolution mapping of DNA binding sites. Elife 6, e21856. 10.7554/eLife.21856 28079019 PMC5310842

[B30] van Steensel B. Henikoff S. (2000). Identification of *in vivo* DNA targets of chromatin proteins using tethered dam methyltransferase. Nat. Biotechnol. 18, 424–428. 10.1038/74487 10748524

[B31] Wang H. Fan Z. Shliaha P. V. Miele M. Hendrickson R. C. Jiang X. (2023). H3K4me3 regulates RNA polymerase II promoter-proximal pause-release. Nature 615, 339–348. 10.1038/s41586-023-05780-8 36859550 PMC9995272

[B32] Yan L. Chen J. Zhu X. Sun J. Wu X. Shen W. (2018). Maternal Huluwa dictates the embryonic body axis through β-catenin in vertebrates. Science 362, eaat1045. 10.1126/science.aat1045 30467143

[B33] Zhang C. Wang M. Li Y. Zhang Y. (2022). Profiling and functional characterization of maternal mRNA translation during mouse maternal-to-zygotic transition. Sci. Adv. 8, eabj3967. 10.1126/sciadv.abj3967 35108058 PMC8809684

[B34] Zhang Y. Liu T. Meyer C. A. Eeckhoute J. Johnson D. S. Bernstein B. E. (2008). Model-based analysis of ChIP-seq (MACS). Genome Biol. 9, R137. 10.1186/gb-2008-9-9-r137 18798982 PMC2592715

[B35] Zheng Q. Xie X. Li Y. Ai C. Pu S. Chen J. (2024). Rapid isolation of stage I oocytes in zebrafish devoid of granulosa cells. J. Vis. Exp 209, e66458. 10.3791/66458 39141564

