# Supplementary material for: Low-input CUT&Tag for efficient epigenomic profiling of zebrafish stage I oocytes
Source: Front Cell Dev Biol. 2024 Dec 4;12:1475912. doi: 10.3389/fcell.2024.1475912 (PMC11652594; doi:10.3389/fcell.2024.1475912)
Supplement: Supplementary file 1 [file DataSheet1.pdf]

1. Material

Reagents

- The Hyperactive Universal CUT&Tag Assay Kit for Illumina (Vazyme, TD903) was used to optimize and improve the CUT&Tag method for zebrafish stage I oocytes. For an input of 1,000 to 3,000 oocytes, the reaction system achieves optimal results when using half the volumes recommended in the kit's instruction manual. The kit components are detailed in Table 1.

Table 1. The composition of Hyperactive Universal CUT&Tag Assay Kit for IIIumina

**Components**		**TD903-01**
BOX1	ConA Beads	130 μL
	DNA Extract Beads	250 μL
	Buffer L/B	2×750 μL
	Buffer WA	1.5 mL
	Buffer WB	1.5 mL
BOX2	pA/G-Tnp(2 μM)	24 μL
	5% Digitonin	150 μL
	10×Binding Buffer	400 μL
	NE Buffer	1.2 mL
	10×Wash Buffer	2×1 mL
	10×Dig-300 Buffer	1.2 mL
	Antibody Buffer(-)	1 mL
	5×TTBL	150 μL
	Proteinase K	80 μL
	2×CAM	600 μL

- TruePrep® Index Kit V2 for Illumina (Vazyme, TD202)
- Protease Inhibitor Cocktail, EDTA-Free, 100X in DMSO (Bimake, B14001)
- L-15 medium, with L-glutamine (Hyclone, SH30525.01)
- 100% Ethanol (KESHI, CAS 64-17-5)
- Antibody: H3K4me3 rabbit polyclonal antibody (Active Motif, 39060), H3K27Ac rabbit polyclonal antibody (abcam, ab4729), H3K27me3 rabbit polyclonal antibody (Diagenode, C15410069), H3K4me1 rabbit polyclonal antibody (Active Motif, 39498).
- Secondary antibody: Goat anti-rabbit IgG H&L (Vazyme, Ab206-01-AA)

- Qubit dsDNA HS Assay Kit (Invitrogen, Q32851)
- VAHTS DNA Clean Beads（Vazyme, N411）
- 1 × TAE, containing 40 mM Tris, 20 mM Acetate, 1 mM EDTA
- 10 × DNA loading buffer (Vazyme, P505-d1-AD)
- DL5000 DNA ladder (Genesand, Biotech, SM812)

Equipment

- magnetic separator, Bimake
- Stereomicroscope, Motic， SMZ-161
- Fluorescence microscope, Zeiss， Axio Zoom.V16
- Tube rotator mixer, Kylin Bell, BE-1100
- Palm Micro Centrifuge, DLAB, D1008
- 10/200/1000 μL Low adsorption pipette tips, Labsellect, T-0010-LR-R-S/ T-0200-LR-R-S/ T-1000-LR-R-S
- Qubit 4 Fluorometer, Invitrogen, Q33226
- 0.2 mL Thin-wall PCR tubes, Axygen, PCR-02-C
- PCR Thermocycler, BIOER, LifeECO
- Gel Electrophoresis System, JUNYI, JY300E and JY-SPCT

Reagent setup

- Binding Buffer: Mix 15 μL of 10× Binding Buffer with ddH_2O to a final volume of 150 μL, and mix thoroughly.
- Wash Buffer: Mix 80 μL of 10× Wash Buffer with 8 μL of 100× Protease Inhibitor (PI) and 712 μL of ddH_2O, then mix thoroughly.
- Antibody Buffer: Mix 25 μL of Antibody Buffer (-) with 0.25 μL of 5% Digitonin, mix thoroughly, and place on ice to pre-cool.
- Dig-wash Buffer: Take away 495 μL of Wash Buffer, mix with 5 μL of 5% Digitonin.
- Dig-300 Buffer: Mix 50 μL of 10× Dig-300 Buffer with 1 μL of 5% Digitonin, 5 μL of 100× Protease Inhibitor (PI), and 444 μL of ddH_2O, then mix thoroughly.

Caution: All reagents should be thoroughly mixed before use. To prevent degradation, digitonin should be protected from extended exposure to room temperature. Reagents

in this experiment are prepared for single-sample use. For initial use of Buffer WA and Buffer WB, add the specified volume of 100% ethanol as indicated on the bottle label, and mark the bottle accordingly.

2. Procedure

ConA Bead Treatment

4.1 Mix the ConA Beads thoroughly using a pipette. Transfer 5 µL of ConA Beads to a 200 µL tube, add 50 µL Binding Buffer, mix thoroughly. Place the tube on a magnetic separator and waiting the mixture becomes clear (approximately 2 minutes), discard the supernatant.

Caution: Prior to use, allow the ConA beads equilibrate to room temperature.

4.2 Remove the tube from magnetic separator, add 50 µL Binding Buffer to resuspend the ConA Beads, mix thoroughly and place the tube back on the magnetic separator, waiting the mixture becomes clear then discard the supernatant.

4.3 Add 5 µL Binding Buffer to resuspend the ConA Beads.

Collection of Zebrafish Stage I Oocytes

4.4 Collect zebrafish stage I oocytes at room temperature and count approximately 2000 cells per sample. The collected oocytes should be subjected to staining by Hoechst to ensure the absence of granulosa cell contamination (Figure 2).

Caution: It is recommended to use juvenile fish between 5- and 7-weeks post-fertilization (wpf) with an SL ranging from 10 mm to 15 mm for this procedure. Detailed operation can be found in our previous publication.

4.5 Transfer the oocytes to a 200 µL tube. Allow to settle for a few minutes. Remove the supernatant when all oocytes have settled to bottom of the tube as observed under stereomicroscope.

Critical step: Oocytes are extremely fragile and cannot tolerate centrifugation. However, their large size allows them to sediment naturally under gravity, which is preferable to optimize collection efficiency. If centrifugation is required for subsequent steps, a palm

centrifuge at a speed below 100g is recommended. When removing the supernatant, leave a small amount of liquid to keep the oocytes submerged, thereby avoiding rupture due to surface tension.

4.6 Resuspend the cells in 50 μL of Wash Buffer and, once all oocytes have settled at the bottom as confirmed under a stereomicroscope, carefully remove the supernatant.

Caution: Resuspend the oocytes by gently rotating the tube by hand. Avoid using a pipette to mix before the **Tagmentation** steps.

4.7 Repeat step 5.6. and finally, resuspend the cells in 50 μL Wash Buffer for next combination.

Combine Oocytes with ConA Beads

4.8 Transfer 5 μL ConA Beads to the 50 μL zebrafish stage I oocyte. Mix by inverting or gently rotating the tube by hand and incubate on a **rotator mixer** at room temperature for 20 minutes.

Caution: To prevent cell damage, avoid using a pipette to mix the cells and beads. If the oocyte-bead mixture adheres to the tube cap, perform a quick spin using a palm centrifuge at less than 100×g. Proper centrifugation should retrieve liquid from the tube cap while maintaining a uniform suspension of the oocyte-bead complex, without aggregation resulting from the centrifugation process.

Critical step: Oocytes require extended time to bind effectively to the beads. Lengthen the incubation period as needed and evaluate binding efficiency in the following step.

4.9 Place the tube on **magnetic separator** to allow the mixtures to clear and collect the supernatant observed under stereomicroscope.

Critical step: The binding time for oocyte-bead complexes, as well as the capture time using a magnetic separator, is longer than in standard CUT&Tag procedures. To ensure optimal binding and collection, examine the supernatant under a stereomicroscope on a clean dish to identify any unbound cells that may have been discarded.

If numerous bead-bound oocytes are observed in the supernatant, return the supernatant to the tube and place it on the magnetic separator for a few additional minutes before

rechecking. If many oocytes without bead binding are found, repeat the incubation step on a rotator mixer.

Primary Antibody binding

4.10 Add 0.5 μL of primary antibody to 25 μL of pre-cooled Antibody Buffer and mix thoroughly.

Caution: A 1:50 dilution is used for primary antibody. Determine the appropriate dilution ratio through preliminary experiments.

4.11 Resuspend the cell-bead complex in the Antibody Buffer from 5.10 by gently inverting or rotating the tube by hand.

Caution: Gentle manual rotation is recommended to prevent the oocyte-bead complexes from adhering to the tube cap, thereby eliminating the need for additional centrifugation steps.

4.12 Briefly centrifuge (less than 100×g) and incubate at 4°C overnight.

Secondary Antibody Binding

4.13 Add 0.25 μL of secondary antibody to 25 μL of Dig-wash Buffer (1:100 dilution) and mix thoroughly by gently rotating the tube by hand.

4.14 Place the tube on **rotator mixer** at room temperature for 40 minutes.

4.15 Place the tube on **magnetic separator** to allow the mixtures to clear and discard the supernatant.

pA/G-Tnp Binding

4.16 Mix 1 μL pA/G-Tnp in 49 μL Dig-300 Buffer (1:50), mix thoroughly and keep it on ice.

4.17 Briefly centrifuge and place the tube on **magnetic separator** to allow the mixtures to clear and discard the supernatant.

4.18 Add 50 μL Dig-wash Buffer and mix thoroughly, place it on **magnetic separator** and discard the supernatant once the solution clears.

4.19 Repeat steps 5.18 once more (washing twice).

4.20 Add the prepared 50 µL of pA/G-Tnp buffer from step 5.16 to the oocytes-beads complex, mix thoroughly and place the tube on **rotator mixer** at room temperature for 1 h.

Tagmentation

4.21 Mix 5 µL 5× TTBL in 20 µL Dig-300 Buffer and mix thoroughly.

4.22 Briefly centrifuge and place the tube on a **magnetic separator** and discard the supernatant once the solution clears.

4.23 Add 50 µL Dig-300 Buffer and mix thoroughly, place the tube on **magnetic separator** and discard the supernatant once the solution clears.

4.24 Repeat steps 5.23 once more (washing twice).

4.25 Add the prepared 25 µL of 1× TTBL buffer from step 5.21 to the oocytes-beads complex, mix thoroughly and incubate the tube in a thermocycler at 37°C for 30 minutes.

Caution: Do not set the thermocycler heated lid temperature.

DNA Extraction

4.26 Add 50 µL Buffer L/B, 10 µL DNA Extract Beads, and 2.5 µL Proteinase K to the tube, mix by inverting and incubate in a thermocycler at 55°C for 10 minutes. Mix 2-3 times during the incubation.

Caution: Do not set the thermocycler heated lid temperature.

4.27 Briefly centrifuge, place the tube on **magnetic separator** to allow the mixtures to clear and discard the supernatant.

4.28 Add 100 µL WA (ensure ethanol has been added before use), gently vortex then briefly centrifuge. Place the tube on a **magnetic separator** to allow the mixtures to clear and discard the supernatant.

4.29 Add 100 µL WB (ensure ethanol has been added before use), gently vortex then briefly centrifuge. Place the tube on a **magnetic separator** to allow the mixtures to clear and discard the supernatant.

4.30 Repeat step 5.29 once more.

4.31 Air dry until the ethanol evaporates.

Caution: Ensure that the beads are neither too dry nor retaining excess Wash Buffer (WB), as this can impact elution efficiency and affect subsequent library construction. The bead surface can be monitored under light; a change from a glossy to a non-reflective appearance indicates the appropriate level of dryness.

4.32 Remove the tube from **magnetic separator**, add 20 μL ddH_2O, mix gently by pipette and shaking 2-3 times during elution, waiting for 5 minutes.

4.33 Place the tube on a **magnetic separator** to allow the mixtures to clear, transfer 18 μL of the supernatant to a new tube.

PAUSE POINT: The DNA can be stored at -30℃ to -15℃, avoiding repeated freeze-thaw.

Optimizing the PCR Cycles for Library Construction

4.34 Mix the PCR reaction components in a tube on ice according to the table. using the different barcode for each sample.

Table 1 PCR reaction system

Components	Volume
Extracted DNA fragment	1μL
2×CAM	25 μL
N5XX	5 μL
N7XX	5 μL
ddH_2O	14 μL
Total volume	50 μL

4.35 Divide the reaction mixture from step 5.34 equally into three tubes, set the cycle numbers to 19, 22, and 25, respectively, and perform the following reaction in thermocycler.

Table 2 PCR procedure

Temperature	Time	cycle number
72°C	3 min	1

95℃	3 min	1
98℃	10 sec	19/22/25
60℃	5 sec	
72℃	1 min	1
4℃	Hold	

4.36 Take 4.5 μL PCR product from step 5.35 and add 0.5 μL 10× DNA loading buffer. Perform electrophoresis on a 1.5% agarose gel. Determine the size distribution of DNA (Figure 2B).

4.37 Determining the optimal cycle number: the brightness of the DNA bands gradually increases as the cycle number increases, choose the cycle number at which a faint band first appears. If the 19 cycles are used as a reference, the remaining 16 μL DNA template would be mix into a 50 μL PCR system and amplify for 15 cycles. The calculation method is as follows: 2^4=16, 19-4=15. Doubling the template amount or cell number reduces the cycle number by one.

Formal PCR amplification

4.38 Mix the PCR reaction components in a tube on ice according to the table. using the different barcode for each sample:

Components	Volume
Extracted DNA fragment	16 μL
2 ×CAM	25 μL
P5 index	5 μL
P7 index	5 μL
Total volume	50 μL

4.39 Set the cycle Determined by step 5.37 and perform the following reaction in thermocycler.

Temperature	Time	cycle number
72℃	3 min	1
95℃	3 min	1

98℃	10 sec	From step 5.37
60℃	5 sec	
72℃	1 min	1
4℃	Hold	

DNA Library purification

4.40 Bring the VAHTS DNA Clean Beads to room temperature. Vortex to mix thoroughly, then add 100 µL beads to the PCR product. Vortex or pipette up and down 20 times to ensure uniform mixing. Incubate at room temperature for 5 minutes.

4.41 Place the tube on a **magnetic separator** to allow the mixtures to clear and discard the supernatant.

4.42 Keep the PCR tube on the **magnetic separator** and add 200 µL freshly prepared 80% ethanol to wash the beads. Incubate at room temperature for 30 seconds and then carefully remove the supernatant.

4.43 Repeat the step 5.42 once.

4.44 Keep the PCR tube on the **magnetic separator** and air-dry for 3-5 minutes.

Caution: Overdrying can reduce recovery of DNA, Beads can be observed under a light when their surface gloss changes from bright to non-reflective.

4.45 Once the beads no longer appear glossy, remove the tube from the **magnetic separator** and add 20 µL ddH2O and pipette up and down 10 times.

4.46 Incubate at room temperature for 5 minutes. Briefly centrifuge and place it on the **magnetic separator** until the solution clears.

4.47 Carefully transfer 18 µL supernatant to a new 1.5 mL tube. Store at -30°C to -15°C for long-term storage, avoiding repeated freeze-thaw cycles.

Library Electrophoresis and Quantification

4.48 Perform agarose gel electrophoresis on the library to preliminarily assess the distribution and yield. CUT&Tag libraries typically show a smear between 200-1000

bp. For histone libraries, a high-quality library will display a nucleosome periodicity pattern (Figure 2C).

4.49 Quantify the library using Qubit, qPCR, or the Agilent 2100 Bioanalyzer. If the pre-amplification experiment was successful, the purified amplification library can be sent directly for sequencing and standard library quality assessment (Figure 2C).

Data processing and analysis

4.50 Data processing can refer to the standard CUT&Tag analysis workflow. All reads were aligned to the zebrafish reference genome (danRer11) using Bowtie2 (version 2.2.2). All unmapped reads, nonuniquely mapped reads, and PCR duplicates were removed. To visualize the signals in the UCSC Genome Browser, each read was extended by 250 bp, and the coverage for each base was counted. Peaks were called using MACS2. The called peaks with weak signals were filtered in the further analysis.
